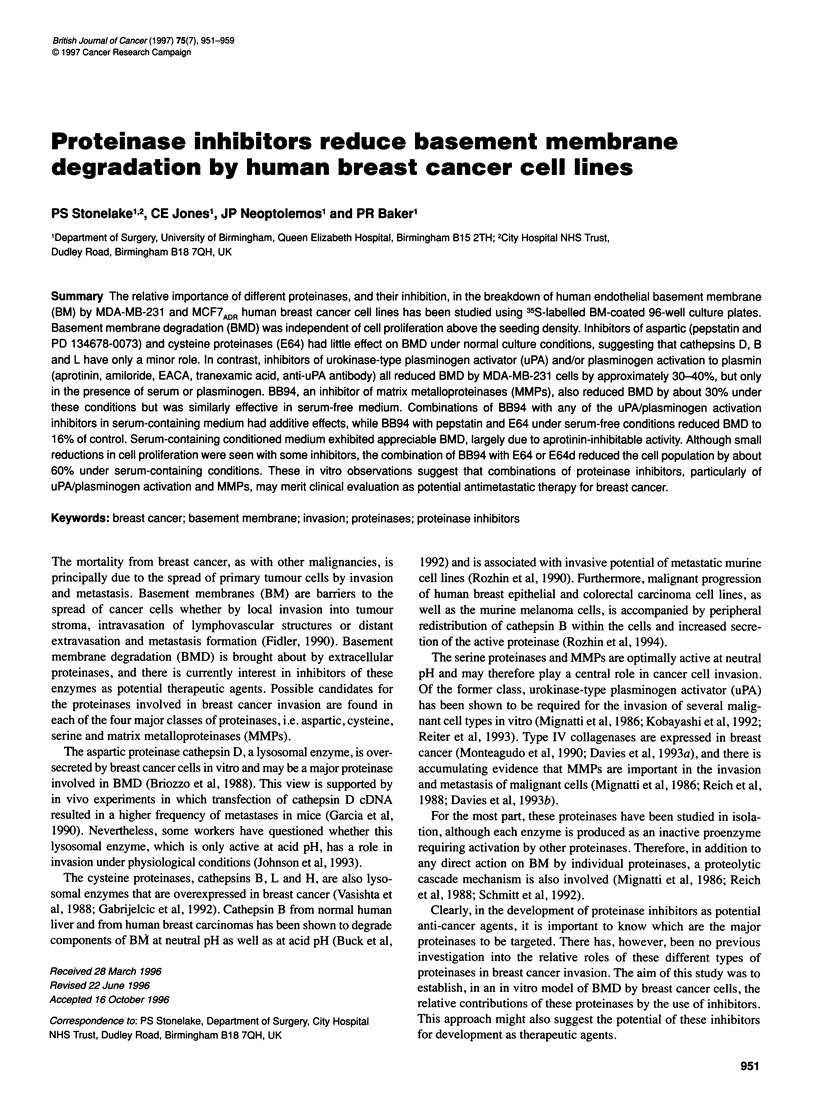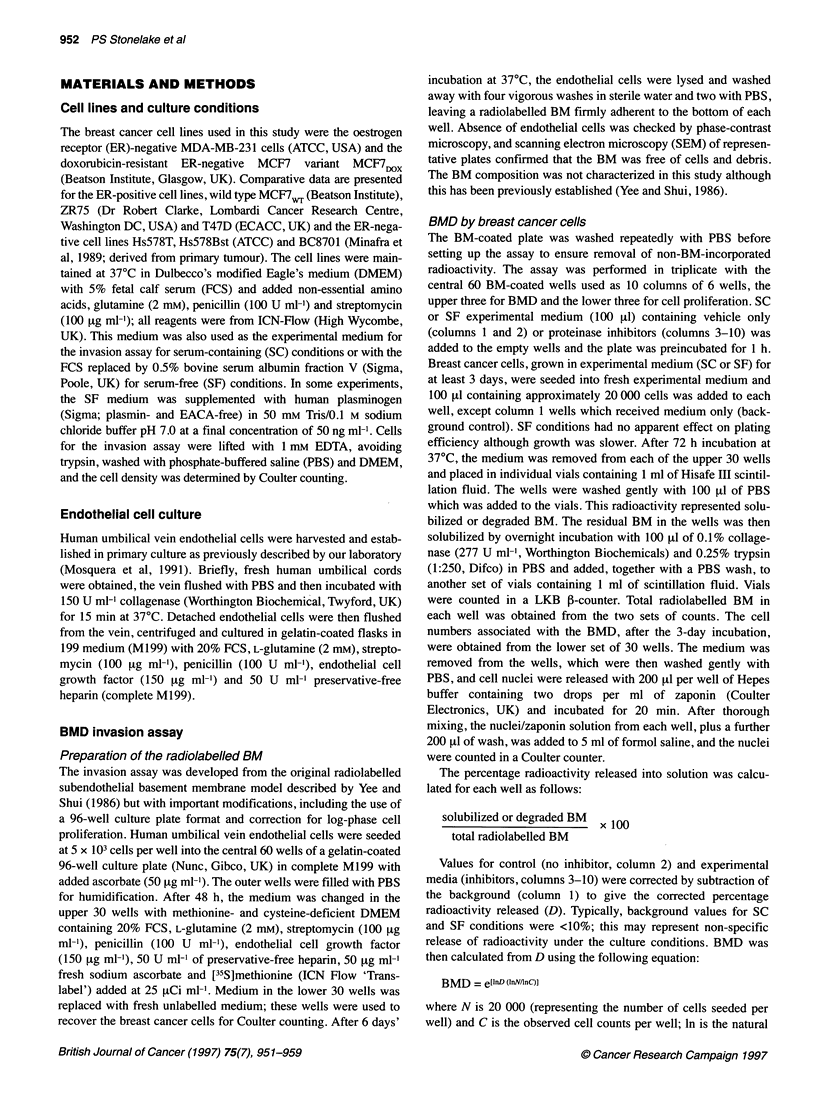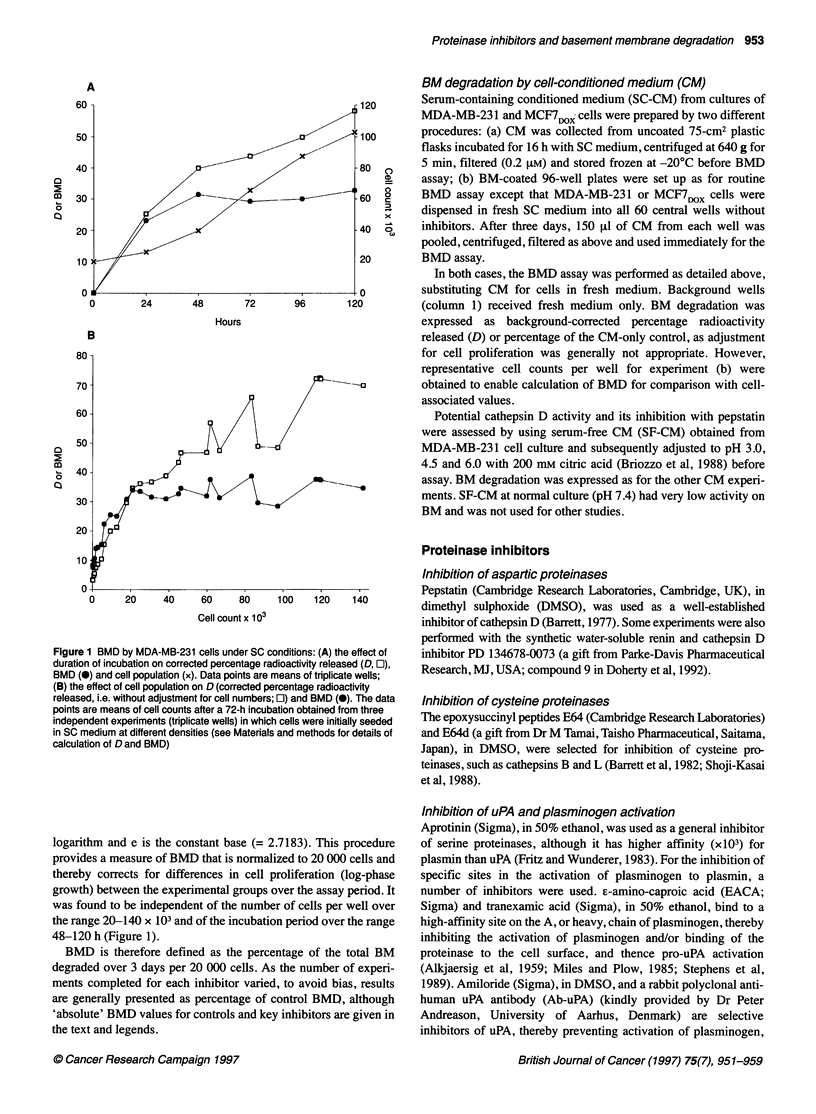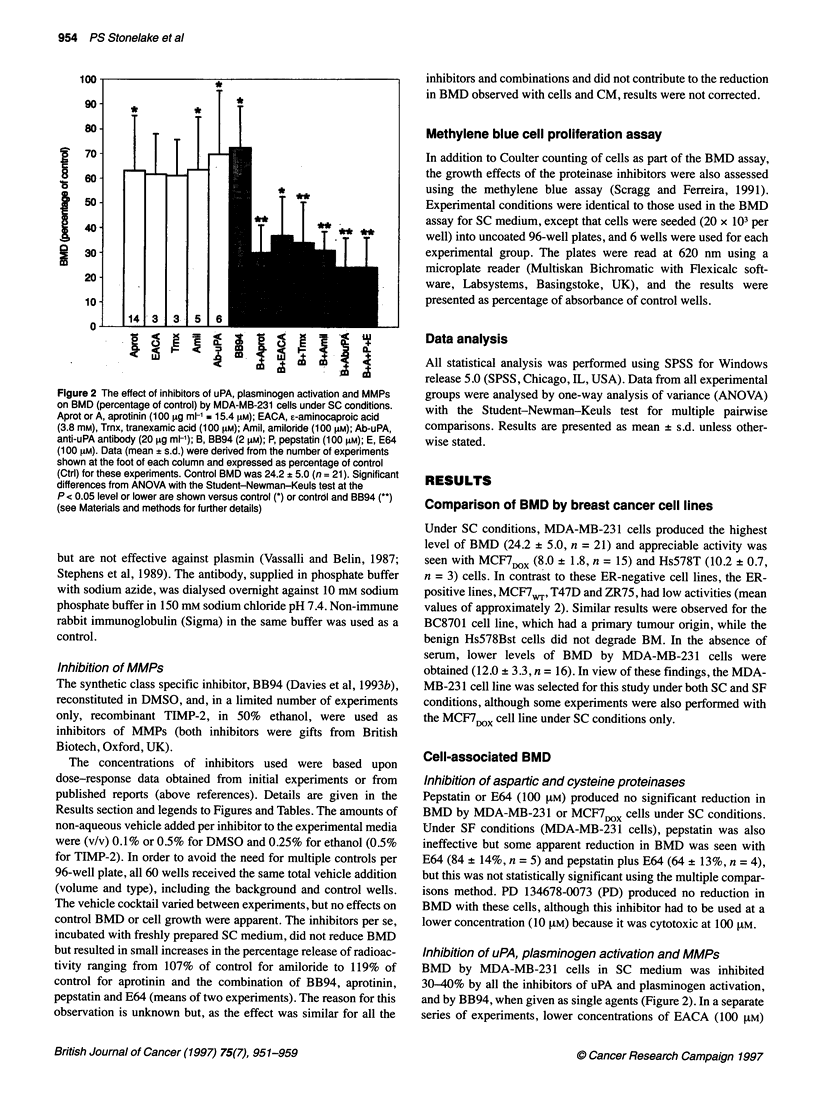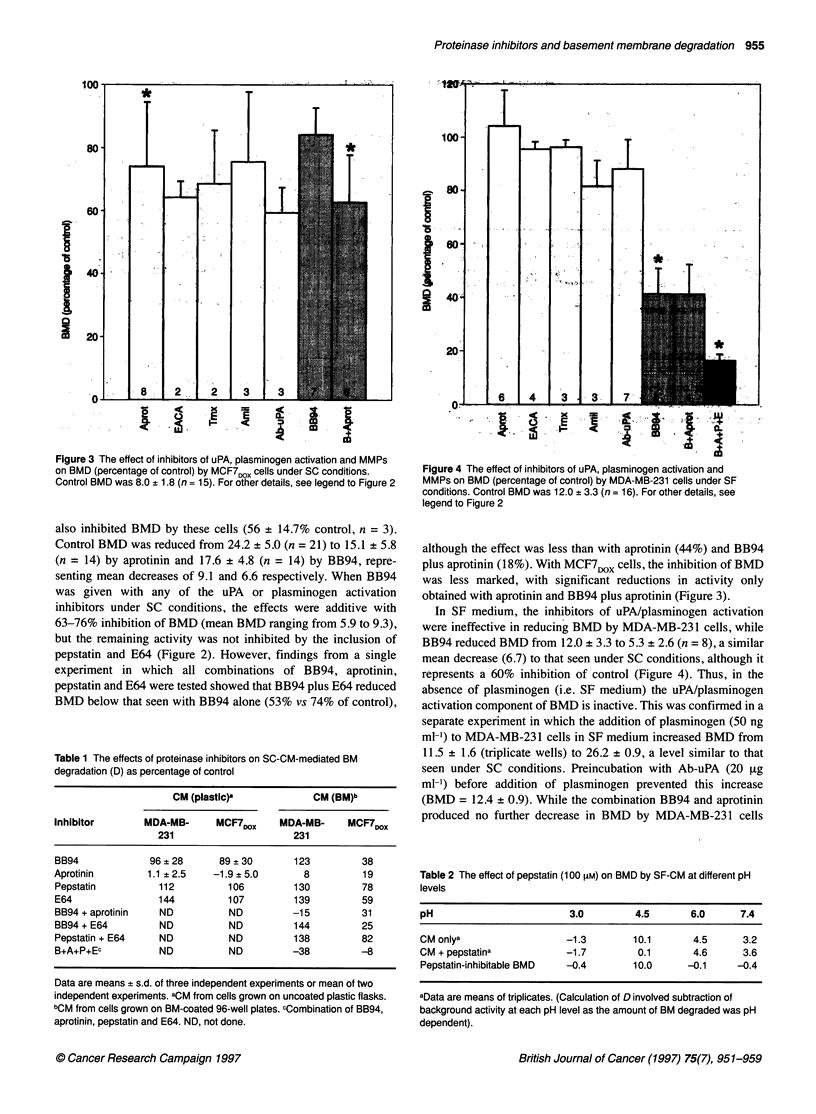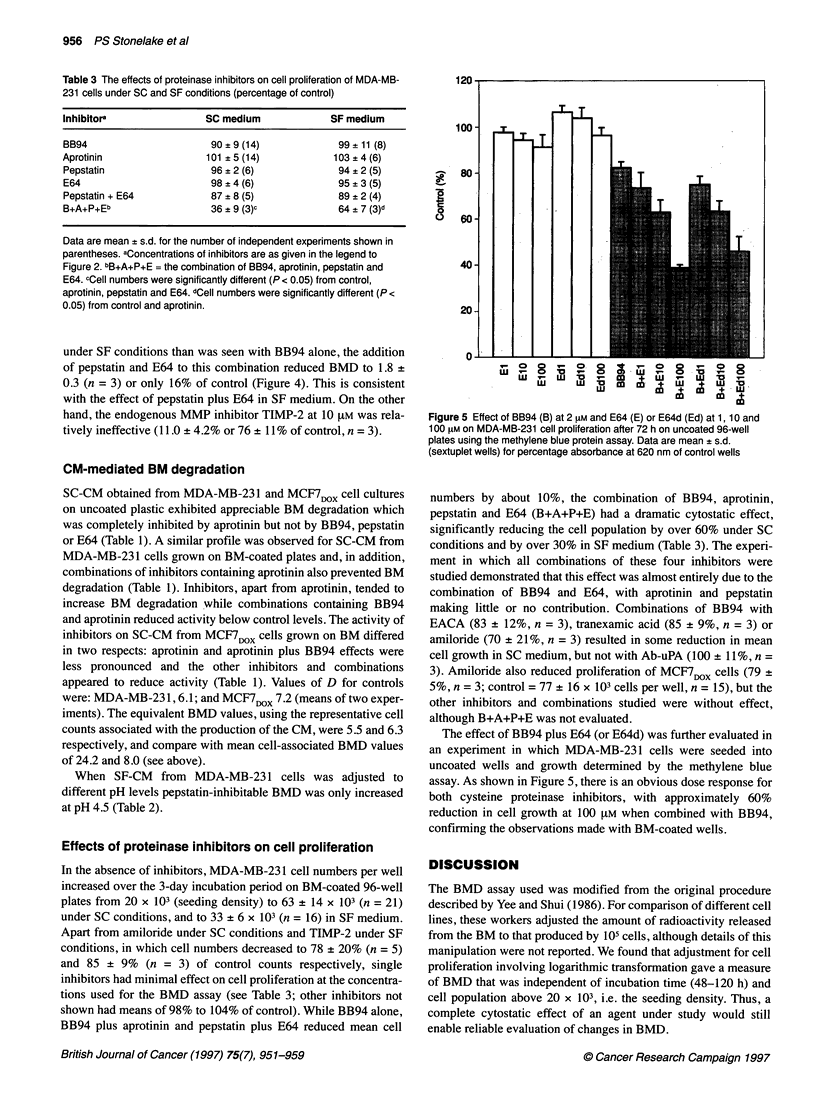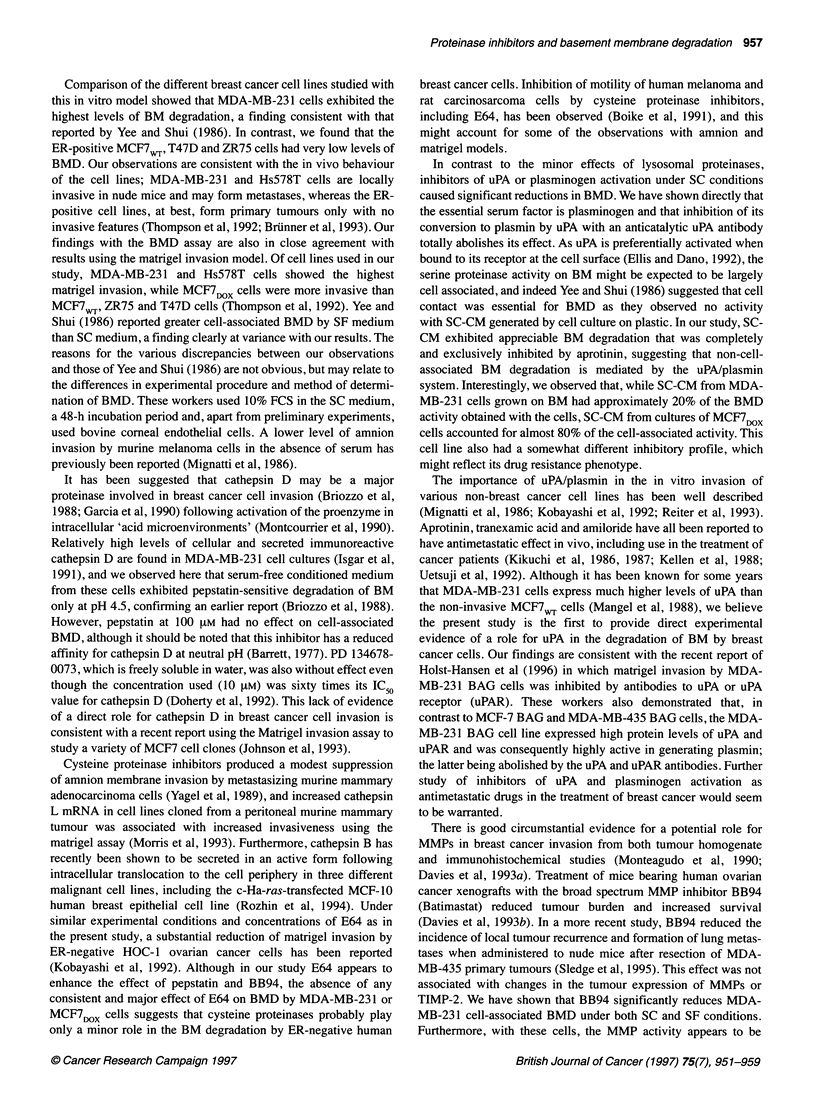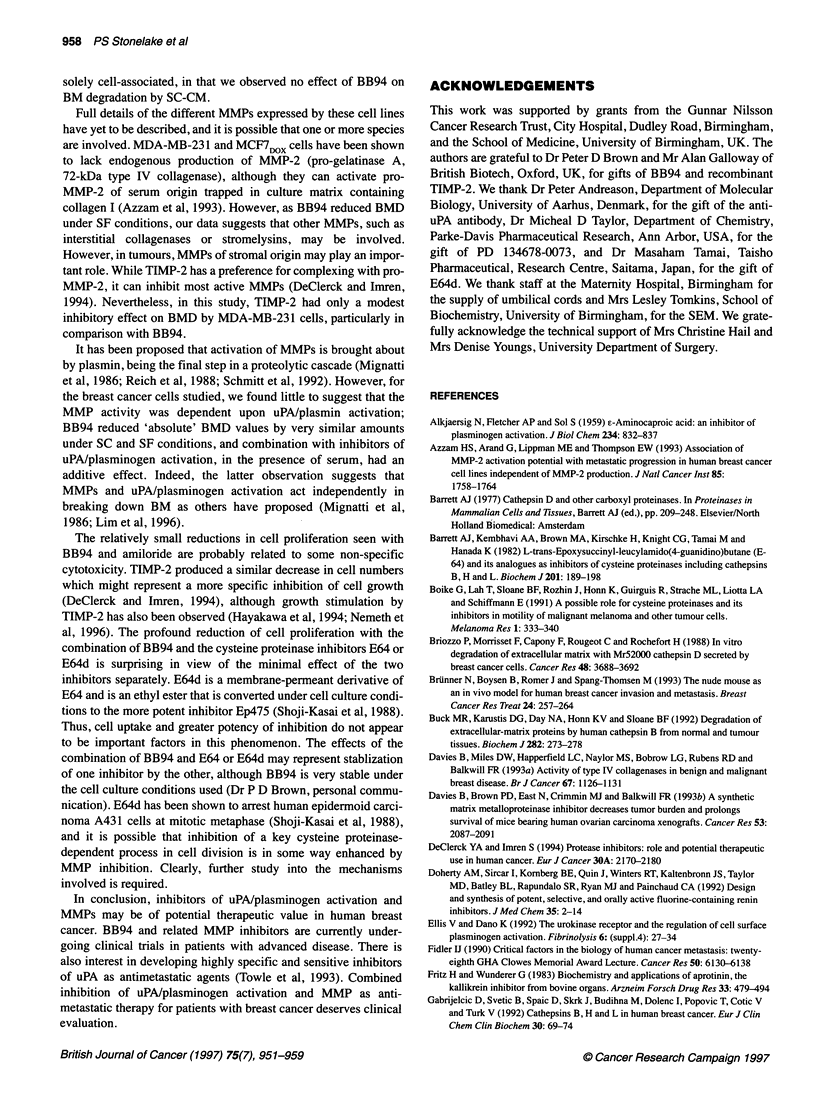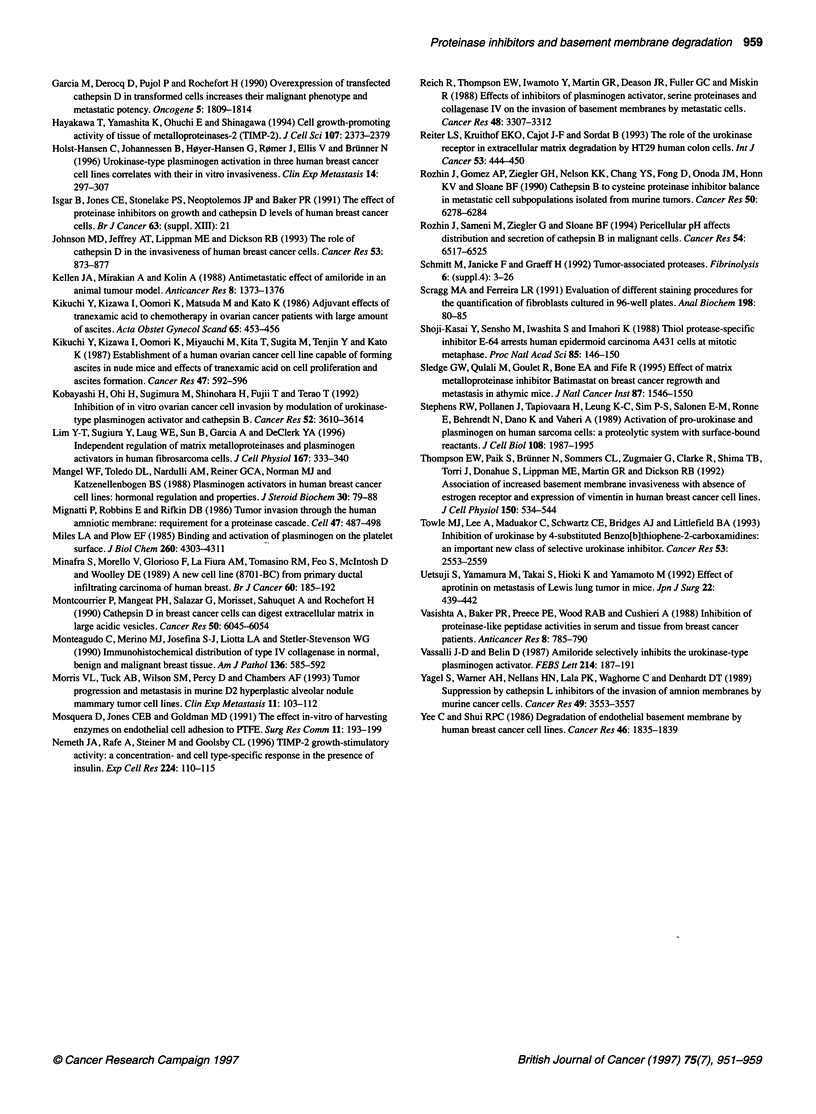# Proteinase inhibitors reduce basement membrane degradation by human breast cancer cell lines.

**DOI:** 10.1038/bjc.1997.166

**Published:** 1997

**Authors:** P. S. Stonelake, C. E. Jones, J. P. Neoptolemos, P. R. Baker

**Affiliations:** Department of Surgery, University of Birmingham, UK.

## Abstract

The relative importance of different proteinases, and their inhibition, in the breakdown of human endothelial basement membrane (BM) by MDA-MB-231 and MCF7ADR human breast cancer cell lines has been studied using 35S-labelled BM-coated 96-well culture plates. Basement membrane degradation (BMD) was independent of cell proliferation above the seeding density. Inhibitors of aspartic (pepstatin and PD 134678-0073) and cysteine proteinases (E64) had little effect on BMD under normal culture conditions, suggesting that cathepsins D, B and L have only a minor role. In contrast, inhibitors of urokinase-type plasminogen activator (uPA) and/or plasminogen activation to plasmin (aprotinin, amiloride, EACA, tranexamic acid, anti-uPA antibody) all reduced BMD by MDA-MB-231 cells by approximately 30-40%, but only in the presence of serum or plasminogen. BB94, an inhibitor of matrix metalloproteinases (MMPs), also reduced BMD by about 30% under these conditions but was similarly effective in serum-free medium. Combinations of BB94 with any of the uPA/plasminogen activation inhibitors in serum-containing medium had additive effects, while BB94 with pepstatin and E64 under serum-free conditions reduced BMD to 16% of control. Serum-containing conditioned medium exhibited appreciable BMD, largely due to aprotinin-inhibitable activity. Although small reductions in cell proliferation were seen with some inhibitors, the combination of BB94 with E64 or E64d reduced the cell population by about 60% under serum-containing conditions. These in vitro observations suggest that combinations of proteinase inhibitors, particularly of uPA/plasminogen activation and MMPs, may merit clinical evaluation as potential antimetastatic therapy for breast cancer.


British Journal of Cancer (1997) 75(7), 951–959
© 1997 Cancer Research Campaign

### Proteinase inhibitors reduce basement membrane degradation by human breast cancer cell lines

PS Stonelake[1,2], CE Jones[1], JP Neoptolemos[1] and PR Baker[1]

[1]Department of Surgery, University of Birmingham, Queen Elizabeth Hospital, Birmingham B15 2TH; [2]City Hospital NHS Trust, Dudley Road, Birmingham B18 7QH, UK

**Summary** The relative importance of different proteinases, and their inhibition, in the breakdown of human endothelial basement membrane (BM) by MDA-MB-231 and $MCF7_{ADR}$ human breast cancer cell lines has been studied using $^{35}S$-labelled BM-coated 96-well culture plates. Basement membrane degradation (BMD) was independent of cell proliferation above the seeding density. Inhibitors of aspartic (pepstatin and PD 134678-0073) and cysteine proteinases (E64) had little effect on BMD under normal culture conditions, suggesting that cathepsins D, B and L have only a minor role. In contrast, inhibitors of urokinase-type plasminogen activator (uPA) and/or plasminogen activation to plasmin (aprotinin, amiloride, EACA, tranexamic acid, anti-uPA antibody) all reduced BMD by MDA-MB-231 cells by approximately 30–40%, but only in the presence of serum or plasminogen. BB94, an inhibitor of matrix metalloproteinases (MMPs), also reduced BMD by about 30% under these conditions but was similarly effective in serum-free medium. Combinations of BB94 with any of the uPA/plasminogen activation inhibitors in serum-containing medium had additive effects, while BB94 with pepstatin and E64 under serum-free conditions reduced BMD to 16% of control. Serum-containing conditioned medium exhibited appreciable BMD, largely due to aprotinin-inhibitable activity. Although small reductions in cell proliferation were seen with some inhibitors, the combination of BB94 with E64 or E64d reduced the cell population by about 60% under serum-containing conditions. These in vitro observations suggest that combinations of proteinase inhibitors, particularly of uPA/plasminogen activation and MMPs, may merit clinical evaluation as potential antimetastatic therapy for breast cancer.

**Keywords:** breast cancer; basement membrane; invasion; proteinases; proteinase inhibitors

The mortality from breast cancer, as with other malignancies, is principally due to the spread of primary tumour cells by invasion and metastasis. Basement membranes (BM) are barriers to the spread of cancer cells whether by local invasion into tumour stroma, intravasation of lymphovascular structures or distant extravasation and metastasis formation (Fidler, 1990). Basement membrane degradation (BMD) is brought about by extracellular proteinases, and there is currently interest in inhibitors of these enzymes as potential therapeutic agents. Possible candidates for the proteinases involved in breast cancer invasion are found in each of the four major classes of proteinases, i.e. aspartic, cysteine, serine and matrix metalloproteinases (MMPs).

The aspartic proteinase cathepsin D, a lysosomal enzyme, is over-secreted by breast cancer cells in vitro and may be a major proteinase involved in BMD (Briozzo et al, 1988). This view is supported by in vivo experiments in which transfection of cathepsin D cDNA resulted in a higher frequency of metastases in mice (Garcia et al, 1990). Nevertheless, some workers have questioned whether this lysosomal enzyme, which is only active at acid pH, has a role in invasion under physiological conditions (Johnson et al, 1993).

The cysteine proteinases, cathepsins B, L and H, are also lysosomal enzymes that are overexpressed in breast cancer (Vasishta et al, 1988; Gabrijelcic et al, 1992). Cathepsin B from normal human liver and from human breast carcinomas has been shown to degrade components of BM at neutral pH as well as at acid pH (Buck et al, 1992) and is associated with invasive potential of metastatic murine cell lines (Rozhin et al, 1990). Furthermore, malignant progression of human breast epithelial and colorectal carcinoma cell lines, as well as the murine melanoma cells, is accompanied by peripheral redistribution of cathepsin B within the cells and increased secretion of the active proteinase (Rozhin et al, 1994).

The serine proteinases and MMPs are optimally active at neutral pH and may therefore play a central role in cancer cell invasion. Of the former class, urokinase-type plasminogen activator (uPA) has been shown to be required for the invasion of several malignant cell types in vitro (Mignatti et al, 1986; Kobayashi et al, 1992; Reiter et al, 1993). Type IV collagenases are expressed in breast cancer (Monteagudo et al, 1990; Davies et al, 1993*a*), and there is accumulating evidence that MMPs are important in the invasion and metastasis of malignant cells (Mignatti et al, 1986; Reich et al, 1988; Davies et al, 1993*b*).

For the most part, these proteinases have been studied in isolation, although each enzyme is produced as an inactive proenzyme requiring activation by other proteinases. Therefore, in addition to any direct action on BM by individual proteinases, a proteolytic cascade mechanism is also involved (Mignatti et al, 1986; Reich et al, 1988; Schmitt et al, 1992).

Clearly, in the development of proteinase inhibitors as potential anti-cancer agents, it is important to know which are the major proteinases to be targeted. There has, however, been no previous investigation into the relative roles of these different types of proteinases in breast cancer invasion. The aim of this study was to establish, in an in vitro model of BMD by breast cancer cells, the relative contributions of these proteinases by the use of inhibitors. This approach might also suggest the potential of these inhibitors for development as therapeutic agents.

*Received 28 March 1996*
*Revised 22 June 1996*
*Accepted 16 October 1996*

*Correspondence to:* PS Stonelake, Department of Surgery, City Hospital NHS Trust, Dudley Road, Birmingham B18 7QH, UK

### MATERIALS AND METHODS

### Cell lines and culture conditions

The breast cancer cell lines used in this study were the oestrogen receptor (ER)-negative MDA-MB-231 cells (ATCC, USA) and the doxorubicin-resistant ER-negative MCF7 variant $MCF7_{DOX}$ (Beatson Institute, Glasgow, UK). Comparative data are presented for the ER-positive cell lines, wild type $MCF7_{WT}$ (Beatson Institute), ZR75 (Dr Robert Clarke, Lombardi Cancer Research Centre, Washington DC, USA) and T47D (ECACC, UK) and the ER-negative cell lines Hs578T, Hs578Bst (ATCC) and BC8701 (Minafra et al, 1989; derived from primary tumour). The cell lines were maintained at 37°C in Dulbecco's modified Eagle's medium (DMEM) with 5% fetal calf serum (FCS) and added non-essential amino acids, glutamine (2 mM), penicillin (100 U $ml^{-1}$) and streptomycin (100 µg $ml^{-1}$); all reagents were from ICN-Flow (High Wycombe, UK). This medium was also used as the experimental medium for the invasion assay for serum-containing (SC) conditions or with the FCS replaced by 0.5% bovine serum albumin fraction V (Sigma, Poole, UK) for serum-free (SF) conditions. In some experiments, the SF medium was supplemented with human plasminogen (Sigma; plasmin- and EACA-free) in 50 mM Tris/0.1 M sodium chloride buffer pH 7.0 at a final concentration of 50 ng $ml^{-1}$. Cells for the invasion assay were lifted with 1 mM EDTA, avoiding trypsin, washed with phosphate-buffered saline (PBS) and DMEM, and the cell density was determined by Coulter counting.

### Endothelial cell culture

Human umbilical vein endothelial cells were harvested and established in primary culture as previously described by our laboratory (Mosquera et al, 1991). Briefly, fresh human umbilical cords were obtained, the vein flushed with PBS and then incubated with 150 U $ml^{-1}$ collagenase (Worthington Biochemical, Twyford, UK) for 15 min at 37°C. Detached endothelial cells were then flushed from the vein, centrifuged and cultured in gelatin-coated flasks in 199 medium (M199) with 20% FCS, L-glutamine (2 mM), streptomycin (100 µg $ml^{-1}$), penicillin (100 U $ml^{-1}$), endothelial cell growth factor (150 µg $ml^{-1}$) and 50 U $ml^{-1}$ preservative-free heparin (complete M199).

### BMD invasion assay

#### *Preparation of the radiolabelled BM*

The invasion assay was developed from the original radiolabelled subendothelial basement membrane model described by Yee and Shui (1986) but with important modifications, including the use of a 96-well culture plate format and correction for log-phase cell proliferation. Human umbilical vein endothelial cells were seeded at $5 \times 10^3$ cells per well into the central 60 wells of a gelatin-coated 96-well culture plate (Nunc, Gibco, UK) in complete M199 with added ascorbate (50 µg $ml^{-1}$). The outer wells were filled with PBS for humidification. After 48 h, the medium was changed in the upper 30 wells with methionine- and cysteine-deficient DMEM containing 20% FCS, L-glutamine (2 mM), streptomycin (100 µg $ml^{-1}$), penicillin (100 U $ml^{-1}$), endothelial cell growth factor (150 µg $ml^{-1}$), 50 U $ml^{-1}$ of preservative-free heparin, 50 µg $ml^{-1}$ fresh sodium ascorbate and [$^{35}$S]methionine (ICN Flow 'Translabel') added at 25 µCi $ml^{-1}$. Medium in the lower 30 wells was replaced with fresh unlabelled medium; these wells were used to recover the breast cancer cells for Coulter counting. After 6 days' incubation at 37°C, the endothelial cells were lysed and washed away with four vigorous washes in sterile water and two with PBS, leaving a radiolabelled BM firmly adherent to the bottom of each well. Absence of endothelial cells was checked by phase-contrast microscopy, and scanning electron microscopy (SEM) of representative plates confirmed that the BM was free of cells and debris. The BM composition was not characterized in this study although this has been previously established (Yee and Shui, 1986).

#### *BMD by breast cancer cells*

The BM-coated plate was washed repeatedly with PBS before setting up the assay to ensure removal of non-BM-incorporated radioactivity. The assay was performed in triplicate with the central 60 BM-coated wells used as 10 columns of 6 wells, the upper three for BMD and the lower three for cell proliferation. SC or SF experimental medium (100 µl) containing vehicle only (columns 1 and 2) or proteinase inhibitors (columns 3–10) was added to the empty wells and the plate was preincubated for 1 h. Breast cancer cells, grown in experimental medium (SC or SF) for at least 3 days, were seeded into fresh experimental medium and 100 µl containing approximately 20 000 cells was added to each well, except column 1 wells which received medium only (background control). SF conditions had no apparent effect on plating efficiency although growth was slower. After 72 h incubation at 37°C, the medium was removed from each of the upper 30 wells and placed in individual vials containing 1 ml of Hisafe III scintillation fluid. The wells were washed gently with 100 µl of PBS which was added to the vials. This radioactivity represented solubilized or degraded BM. The residual BM in the wells was then solubilized by overnight incubation with 100 µl of 0.1% collagenase (277 U $ml^{-1}$, Worthington Biochemicals) and 0.25% trypsin (1:250, Difco) in PBS and added, together with a PBS wash, to another set of vials containing 1 ml of scintillation fluid. Vials were counted in a LKB β-counter. Total radiolabelled BM in each well was obtained from the two sets of counts. The cell numbers associated with the BMD, after the 3-day incubation, were obtained from the lower set of 30 wells. The medium was removed from the wells, which were then washed gently with PBS, and cell nuclei were released with 200 µl per well of Hepes buffer containing two drops per ml of zaponin (Coulter Electronics, UK) and incubated for 20 min. After thorough mixing, the nuclei/zaponin solution from each well, plus a further 200 µl of wash, was added to 5 ml of formol saline, and the nuclei were counted in a Coulter counter.

The percentage radioactivity released into solution was calculated for each well as follows:

$$\frac{\text{solubilized or degraded BM}}{\text{total radiolabelled BM}} \times 100$$

Values for control (no inhibitor, column 2) and experimental media (inhibitors, columns 3–10) were corrected by subtraction of the background (column 1) to give the corrected percentage radioactivity released (*D*). Typically, background values for SC and SF conditions were <10%; this may represent non-specific release of radioactivity under the culture conditions. BMD was then calculated from *D* using the following equation:

$$\text{BMD} = e^{[\ln D\,(\ln N/\ln C)]}$$

where *N* is 20 000 (representing the number of cells seeded per well) and *C* is the observed cell counts per well; ln is the natural

© Cancer Research Campaign 1997

**Figure 1** BMD by MDA-MB-231 cells under SC conditions: (**A**) the effect of duration of incubation on corrected percentage radioactivity released (*D*, □), BMD (●) and cell population (x). Data points are means of triplicate wells; (**B**) the effect of cell population on *D* (corrected percentage radioactivity released, i.e. without adjustment for cell numbers; □) and BMD (●). The data points are means of cell counts after a 72-h incubation obtained from three independent experiments (triplicate wells) in which cells were initially seeded in SC medium at different densities (see Materials and methods for details of calculation of *D* and BMD)

logarithm and e is the constant base (= 2.7183). This procedure provides a measure of BMD that is normalized to 20 000 cells and thereby corrects for differences in cell proliferation (log-phase growth) between the experimental groups over the assay period. It was found to be independent of the number of cells per well over the range 20–140 × 10$^3$ and of the incubation period over the range 48–120 h (Figure 1).

BMD is therefore defined as the percentage of the total BM degraded over 3 days per 20 000 cells. As the number of experiments completed for each inhibitor varied, to avoid bias, results are generally presented as percentage of control BMD, although 'absolute' BMD values for controls and key inhibitors are given in the text and legends.

### *BM degradation by cell-conditioned medium (CM)*

Serum-containing conditioned medium (SC-CM) from cultures of MDA-MB-231 and $MCF7_{DOX}$ cells were prepared by two different procedures: (a) CM was collected from uncoated 75-cm$^2$ plastic flasks incubated for 16 h with SC medium, centrifuged at 640 **g** for 5 min, filtered (0.2 μM) and stored frozen at –20°C before BMD assay; (b) BM-coated 96-well plates were set up as for routine BMD assay except that MDA-MB-231 or $MCF7_{DOX}$ cells were dispensed in fresh SC medium into all 60 central wells without inhibitors. After three days, 150 μl of CM from each well was pooled, centrifuged, filtered as above and used immediately for the BMD assay.

In both cases, the BMD assay was performed as detailed above, substituting CM for cells in fresh medium. Background wells (column 1) received fresh medium only. BM degradation was expressed as background-corrected percentage radioactivity released (*D*) or percentage of the CM-only control, as adjustment for cell proliferation was generally not appropriate. However, representative cell counts per well for experiment (b) were obtained to enable calculation of BMD for comparison with cell-associated values.

Potential cathepsin D activity and its inhibition with pepstatin were assessed by using serum-free CM (SF-CM) obtained from MDA-MB-231 cell culture and subsequently adjusted to pH 3.0, 4.5 and 6.0 with 200 mM citric acid (Briozzo et al, 1988) before assay. BM degradation was expressed as for the other CM experiments. SF-CM at normal culture (pH 7.4) had very low activity on BM and was not used for other studies.

### Proteinase inhibitors

### *Inhibition of aspartic proteinases*

Pepstatin (Cambridge Research Laboratories, Cambridge, UK), in dimethyl sulphoxide (DMSO), was used as a well-established inhibitor of cathepsin D (Barrett, 1977). Some experiments were also performed with the synthetic water-soluble renin and cathepsin D inhibitor PD 134678-0073 (a gift from Parke-Davis Pharmaceutical Research, MJ, USA; compound 9 in Doherty et al, 1992).

### *Inhibition of cysteine proteinases*

The epoxysuccinyl peptides E64 (Cambridge Research Laboratories) and E64d (a gift from Dr M Tamai, Taisho Pharmaceutical, Saitama, Japan), in DMSO, were selected for inhibition of cysteine proteinases, such as cathepsins B and L (Barrett et al, 1982; Shoji-Kasai et al, 1988).

### *Inhibition of uPA and plasminogen activation*

Aprotinin (Sigma), in 50% ethanol, was used as a general inhibitor of serine proteinases, although it has higher affinity (×10$^3$) for plasmin than uPA (Fritz and Wunderer, 1983). For the inhibition of specific sites in the activation of plasminogen to plasmin, a number of inhibitors were used. ε-amino-caproic acid (EACA; Sigma) and tranexamic acid (Sigma), in 50% ethanol, bind to a high-affinity site on the A, or heavy, chain of plasminogen, thereby inhibiting the activation of plasminogen and/or binding of the proteinase to the cell surface, and thence pro-uPA activation (Alkjaersig et al, 1959; Miles and Plow, 1985; Stephens et al, 1989). Amiloride (Sigma), in DMSO, and a rabbit polyclonal anti-human uPA antibody (Ab-uPA) (kindly provided by Dr Peter Andreason, University of Aarhus, Denmark) are selective inhibitors of uPA, thereby preventing activation of plasminogen,

**Figure 2** The effect of inhibitors of uPA, plasminogen activation and MMPs on BMD (percentage of control) by MDA-MB-231 cells under SC conditions. Aprot or A, aprotinin (100 μg ml⁻¹ = 15.4 μM); EACA, ε-aminocaproic acid (3.8 mM), Trnx, tranexamic acid (100 μM); Amil, amiloride (100 μM); Ab-uPA, anti-uPA antibody (20 μg ml⁻¹); B, BB94 (2 μM); P, pepstatin (100 μM); E, E64 (100 μM). Data (mean ± s.d.) were derived from the number of experiments shown at the foot of each column and expressed as percentage of control (Ctrl) for these experiments. Control BMD was 24.2 ± 5.0 ($n$ = 21). Significant differences from ANOVA with the Student–Newman–Keuls test at the $P < 0.05$ level or lower are shown versus control (*) or control and BB94 (**) (see Materials and methods for further details)

but are not effective against plasmin (Vassalli and Belin, 1987; Stephens et al, 1989). The antibody, supplied in phosphate buffer with sodium azide, was dialysed overnight against 10 mM sodium phosphate buffer in 150 mM sodium chloride pH 7.4. Non-immune rabbit immunoglobulin (Sigma) in the same buffer was used as a control.

### *Inhibition of MMPs*

The synthetic class specific inhibitor, BB94 (Davies et al, 1993*b*), reconstituted in DMSO, and, in a limited number of experiments only, recombinant TIMP-2, in 50% ethanol, were used as inhibitors of MMPs (both inhibitors were gifts from British Biotech, Oxford, UK).

The concentrations of inhibitors used were based upon dose–response data obtained from initial experiments or from published reports (above references). Details are given in the Results section and legends to Figures and Tables. The amounts of non-aqueous vehicle added per inhibitor to the experimental media were (v/v) 0.1% or 0.5% for DMSO and 0.25% for ethanol (0.5% for TIMP-2). In order to avoid the need for multiple controls per 96-well plate, all 60 wells received the same total vehicle addition (volume and type), including the background and control wells. The vehicle cocktail varied between experiments, but no effects on control BMD or cell growth were apparent. The inhibitors per se, incubated with freshly prepared SC medium, did not reduce BMD but resulted in small increases in the percentage release of radioactivity ranging from 107% of control for amiloride to 119% of control for aprotinin and the combination of BB94, aprotinin, pepstatin and E64 (means of two experiments). The reason for this observation is unknown but, as the effect was similar for all the inhibitors and combinations and did not contribute to the reduction in BMD observed with cells and CM, results were not corrected.

### Methylene blue cell proliferation assay

In addition to Coulter counting of cells as part of the BMD assay, the growth effects of the proteinase inhibitors were also assessed using the methylene blue assay (Scragg and Ferreira, 1991). Experimental conditions were identical to those used in the BMD assay for SC medium, except that cells were seeded (20 × $10^3$ per well) into uncoated 96-well plates, and 6 wells were used for each experimental group. The plates were read at 620 nm using a microplate reader (Multiskan Bichromatic with Flexicalc software, Labsystems, Basingstoke, UK), and the results were presented as percentage of absorbance of control wells.

### Data analysis

All statistical analysis was performed using SPSS for Windows release 5.0 (SPSS, Chicago, IL, USA). Data from all experimental groups were analysed by one-way analysis of variance (ANOVA) with the Student–Newman–Keuls test for multiple pairwise comparisons. Results are presented as mean ± s.d. unless otherwise stated.

### RESULTS

### Comparison of BMD by breast cancer cell lines

Under SC conditions, MDA-MB-231 cells produced the highest level of BMD (24.2 ± 5.0, $n$ = 21) and appreciable activity was seen with $MCF7_{DOX}$ (8.0 ± 1.8, $n$ = 15) and Hs578T (10.2 ± 0.7, $n$ = 3) cells. In contrast to these ER-negative cell lines, the ER-positive lines, $MCF7_{WT}$, T47D and ZR75, had low activities (mean values of approximately 2). Similar results were observed for the BC8701 cell line, which had a primary tumour origin, while the benign Hs578Bst cells did not degrade BM. In the absence of serum, lower levels of BMD by MDA-MB-231 cells were obtained (12.0 ± 3.3, $n$ = 16). In view of these findings, the MDA-MB-231 cell line was selected for this study under both SC and SF conditions, although some experiments were also performed with the $MCF7_{DOX}$ cell line under SC conditions only.

### Cell-associated BMD

### *Inhibition of aspartic and cysteine proteinases*

Pepstatin or E64 (100 μM) produced no significant reduction in BMD by MDA-MB-231 or $MCF7_{DOX}$ cells under SC conditions. Under SF conditions (MDA-MB-231 cells), pepstatin was also ineffective but some apparent reduction in BMD was seen with E64 (84 ± 14%, $n$ = 5) and pepstatin plus E64 (64 ± 13%, $n$ = 4), but this was not statistically significant using the multiple comparisons method. PD 134678-0073 (PD) produced no reduction in BMD with these cells, although this inhibitor had to be used at a lower concentration (10 μM) because it was cytotoxic at 100 μM.

### *Inhibition of uPA, plasminogen activation and MMPs*

BMD by MDA-MB-231 cells in SC medium was inhibited 30–40% by all the inhibitors of uPA and plasminogen activation, and by BB94, when given as single agents (Figure 2). In a separate series of experiments, lower concentrations of EACA (100 μM)

© Cancer Research Campaign 1997

**Figure 3** The effect of inhibitors of uPA, plasminogen activation and MMPs on BMD (percentage of control) by $MCF7_{DOX}$ cells under SC conditions. Control BMD was 8.0 ± 1.8 (*n* = 15). For other details, see legend to Figure 2

**Figure 4** The effect of inhibitors of uPA, plasminogen activation and MMPs on BMD (percentage of control) by MDA-MB-231 cells under SF conditions. Control BMD was 12.0 ± 3.3 (*n* = 16). For other details, see legend to Figure 2

also inhibited BMD by these cells (56 ± 14.7% control, *n* = 3). Control BMD was reduced from 24.2 ± 5.0 (*n* = 21) to 15.1 ± 5.8 (*n* = 14) by aprotinin and 17.6 ± 4.8 (*n* = 14) by BB94, representing mean decreases of 9.1 and 6.6 respectively. When BB94 was given with any of the uPA or plasminogen activation inhibitors under SC conditions, the effects were additive with 63–76% inhibition of BMD (mean BMD ranging from 5.9 to 9.3), but the remaining activity was not inhibited by the inclusion of pepstatin and E64 (Figure 2). However, findings from a single experiment in which all combinations of BB94, aprotinin, pepstatin and E64 were tested showed that BB94 plus E64 reduced BMD below that seen with BB94 alone (53% *vs* 74% of control), although the effect was less than with aprotinin (44%) and BB94 plus aprotinin (18%). With $MCF7_{DOX}$ cells, the inhibition of BMD was less marked, with significant reductions in activity only obtained with aprotinin and BB94 plus aprotinin (Figure 3).

In SF medium, the inhibitors of uPA/plasminogen activation were ineffective in reducing BMD by MDA-MB-231 cells, while BB94 reduced BMD from 12.0 ± 3.3 to 5.3 ± 2.6 (*n* = 8), a similar mean decrease (6.7) to that seen under SC conditions, although it represents a 60% inhibition of control (Figure 4). Thus, in the absence of plasminogen (i.e. SF medium) the uPA/plasminogen activation component of BMD is inactive. This was confirmed in a separate experiment in which the addition of plasminogen (50 ng $ml^{-1}$) to MDA-MB-231 cells in SF medium increased BMD from 11.5 ± 1.6 (triplicate wells) to 26.2 ± 0.9, a level similar to that seen under SC conditions. Preincubation with Ab-uPA (20 μg $ml^{-1}$) before addition of plasminogen prevented this increase (BMD = 12.4 ± 0.9). While the combination BB94 and aprotinin produced no further decrease in BMD by MDA-MB-231 cells

**Table 1** The effects of proteinase inhibitors on SC-CM-mediated BM degradation (D) as percentage of control

| Inhibitor | CM (plastic)[a] | | CM (BM)[b] | |
|---|---|---|---|---|
| | MDA-MB-231 | $MCF7_{DOX}$ | MDA-MB-231 | $MCF7_{DOX}$ |
| BB94 | 96 ± 28 | 89 ± 30 | 123 | 38 |
| Aprotinin | 1.1 ± 2.5 | −1.9 ± 5.0 | 8 | 19 |
| Pepstatin | 112 | 106 | 130 | 78 |
| E64 | 144 | 107 | 139 | 59 |
| BB94 + aprotinin | ND | ND | −15 | 31 |
| BB94 + E64 | ND | ND | 144 | 25 |
| Pepstatin + E64 | ND | ND | 138 | 82 |
| B+A+P+E[c] | ND | ND | −38 | −8 |

Data are means ± s.d. of three independent experiments or mean of two independent experiments. [a]CM from cells grown on uncoated plastic flasks. [b]CM from cells grown on BM-coated 96-well plates. [c]Combination of BB94, aprotinin, pepstatin and E64. ND, not done.

**Table 2** The effect of pepstatin (100 μM) on BMD by SF-CM at different pH levels

| pH | 3.0 | 4.5 | 6.0 | 7.4 |
|---|---|---|---|---|
| CM only[a] | −1.3 | 10.1 | 4.5 | 3.2 |
| CM + pepstatin[a] | −1.7 | 0.1 | 4.6 | 3.6 |
| Pepstatin-inhibitable BMD | −0.4 | 10.0 | −0.1 | −0.4 |

[a]Data are means of triplicates. (Calculation of *D* involved subtraction of background activity at each pH level as the amount of BM degraded was pH dependent).

*© Cancer Research Campaign 1997*

**Table 3** The effects of proteinase inhibitors on cell proliferation of MDA-MB-231 cells under SC and SF conditions (percentage of control)

| Inhibitor[a] | SC medium | SF medium |
|---|---|---|
| BB94 | 90 ± 9 (14) | 99 ± 11 (8) |
| Aprotinin | 101 ± 5 (14) | 103 ± 4 (6) |
| Pepstatin | 96 ± 2 (6) | 94 ± 2 (5) |
| E64 | 98 ± 4 (6) | 95 ± 3 (5) |
| Pepstatin + E64 | 87 ± 8 (5) | 89 ± 2 (4) |
| B+A+P+E[b] | 36 ± 9 (3)[c] | 64 ± 7 (3)[d] |

Data are mean ± s.d. for the number of independent experiments shown in parentheses. [a]Concentrations of inhibitors are as given in the legend to Figure 2. [b]B+A+P+E = the combination of BB94, aprotinin, pepstatin and E64. [c]Cell numbers were significantly different ($P < 0.05$) from control, aprotinin, pepstatin and E64. [d]Cell numbers were significantly different ($P < 0.05$) from control and aprotinin.

under SF conditions than was seen with BB94 alone, the addition of pepstatin and E64 to this combination reduced BMD to 1.8 ± 0.3 ($n$ = 3) or only 16% of control (Figure 4). This is consistent with the effect of pepstatin plus E64 in SF medium. On the other hand, the endogenous MMP inhibitor TIMP-2 at 10 μM was relatively ineffective (11.0 ± 4.2% or 76 ± 11% of control, $n$ = 3).

### CM-mediated BM degradation

SC-CM obtained from MDA-MB-231 and $MCF7_{DOX}$ cell cultures on uncoated plastic exhibited appreciable BM degradation which was completely inhibited by aprotinin but not by BB94, pepstatin or E64 (Table 1). A similar profile was observed for SC-CM from MDA-MB-231 cells grown on BM-coated plates and, in addition, combinations of inhibitors containing aprotinin also prevented BM degradation (Table 1). Inhibitors, apart from aprotinin, tended to increase BM degradation while combinations containing BB94 and aprotinin reduced activity below control levels. The activity of inhibitors on SC-CM from $MCF7_{DOX}$ cells grown on BM differed in two respects: aprotinin and aprotinin plus BB94 effects were less pronounced and the other inhibitors and combinations appeared to reduce activity (Table 1). Values of $D$ for controls were: MDA-MB-231, 6.1; and $MCF7_{DOX}$ 7.2 (means of two experiments). The equivalent BMD values, using the representative cell counts associated with the production of the CM, were 5.5 and 6.3 respectively, and compare with mean cell-associated BMD values of 24.2 and 8.0 (see above).

When SF-CM from MDA-MB-231 cells was adjusted to different pH levels pepstatin-inhibitable BMD was only increased at pH 4.5 (Table 2).

### Effects of proteinase inhibitors on cell proliferation

In the absence of inhibitors, MDA-MB-231 cell numbers per well increased over the 3-day incubation period on BM-coated 96-well plates from 20 × $10^3$ (seeding density) to 63 ± 14 × $10^3$ ($n$ = 21) under SC conditions, and to 33 ± 6 × $10^3$ ($n$ = 16) in SF medium. Apart from amiloride under SC conditions and TIMP-2 under SF conditions, in which cell numbers decreased to 78 ± 20% ($n$ = 5) and 85 ± 9% ($n$ = 3) of control counts respectively, single inhibitors had minimal effect on cell proliferation at the concentrations used for the BMD assay (see Table 3; other inhibitors not shown had means of 98% to 104% of control). While BB94 alone, BB94 plus aprotinin and pepstatin plus E64 reduced mean cell

**Figure 5** Effect of BB94 (B) at 2 μM and E64 (E) or E64d (Ed) at 1, 10 and 100 μM on MDA-MB-231 cell proliferation after 72 h on uncoated 96-well plates using the methylene blue protein assay. Data are mean ± s.d. (sextuplet wells) for percentage absorbance at 620 nm of control wells numbers by about 10%, the combination of BB94, aprotinin, pepstatin and E64 (B+A+P+E) had a dramatic cytostatic effect, significantly reducing the cell population by over 60% under SC conditions and by over 30% in SF medium (Table 3). The experiment in which all combinations of these four inhibitors were studied demonstrated that this effect was almost entirely due to the combination of BB94 and E64, with aprotinin and pepstatin making little or no contribution. Combinations of BB94 with EACA (83 ± 12%, $n$ = 3), tranexamic acid (85 ± 9%, $n$ = 3) or amiloride (70 ± 21%, $n$ = 3) resulted in some reduction in mean cell growth in SC medium, but not with Ab-uPA (100 ± 11%, $n$ = 3). Amiloride also reduced proliferation of $MCF7_{DOX}$ cells (79 ± 5%, $n$ = 3; control = 77 ± 16 × $10^3$ cells per well, $n$ = 15), but the other inhibitors and combinations studied were without effect, although B+A+P+E was not evaluated.

The effect of BB94 plus E64 (or E64d) was further evaluated in an experiment in which MDA-MB-231 cells were seeded into uncoated wells and growth determined by the methylene blue assay. As shown in Figure 5, there is an obvious dose response for both cysteine proteinase inhibitors, with approximately 60% reduction in cell growth at 100 μM when combined with BB94, confirming the observations made with BM-coated wells.

### DISCUSSION

The BMD assay used was modified from the original procedure described by Yee and Shui (1986). For comparison of different cell lines, these workers adjusted the amount of radioactivity released from the BM to that produced by $10^5$ cells, although details of this manipulation were not reported. We found that adjustment for cell proliferation involving logarithmic transformation gave a measure of BMD that was independent of incubation time (48–120 h) and cell population above 20 × $10^3$, i.e. the seeding density. Thus, a complete cytostatic effect of an agent under study would still enable reliable evaluation of changes in BMD.

© Cancer Research Campaign 1997

Comparison of the different breast cancer cell lines studied with this in vitro model showed that MDA-MB-231 cells exhibited the highest levels of BM degradation, a finding consistent with that reported by Yee and Shui (1986). In contrast, we found that the ER-positive $MCF7_{WT}$, T47D and ZR75 cells had very low levels of BMD. Our observations are consistent with the in vivo behaviour of the cell lines; MDA-MB-231 and Hs578T cells are locally invasive in nude mice and may form metastases, whereas the ER-positive cell lines, at best, form primary tumours only with no invasive features (Thompson et al, 1992; Brünner et al, 1993). Our findings with the BMD assay are also in close agreement with results using the matrigel invasion model. Of cell lines used in our study, MDA-MB-231 and Hs578T cells showed the highest matrigel invasion, while $MCF7_{DOX}$ cells were more invasive than $MCF7_{WT}$, ZR75 and T47D cells (Thompson et al, 1992). Yee and Shui (1986) reported greater cell-associated BMD by SF medium than SC medium, a finding clearly at variance with our results. The reasons for the various discrepancies between our observations and those of Yee and Shui (1986) are not obvious, but may relate to the differences in experimental procedure and method of determination of BMD. These workers used 10% FCS in the SC medium, a 48-h incubation period and, apart from preliminary experiments, used bovine corneal endothelial cells. A lower level of amnion invasion by murine melanoma cells in the absence of serum has previously been reported (Mignatti et al, 1986).

It has been suggested that cathepsin D may be a major proteinase involved in breast cancer cell invasion (Briozzo et al, 1988; Garcia et al, 1990) following activation of the proenzyme in intracellular 'acid microenvironments' (Montcourrier et al, 1990). Relatively high levels of cellular and secreted immunoreactive cathepsin D are found in MDA-MB-231 cell cultures (Isgar et al, 1991), and we observed here that serum-free conditioned medium from these cells exhibited pepstatin-sensitive degradation of BM only at pH 4.5, confirming an earlier report (Briozzo et al, 1988). However, pepstatin at 100 μM had no effect on cell-associated BMD, although it should be noted that this inhibitor has a reduced affinity for cathepsin D at neutral pH (Barrett, 1977). PD 134678-0073, which is freely soluble in water, was also without effect even though the concentration used (10 μM) was sixty times its $IC_{50}$ value for cathepsin D (Doherty et al, 1992). This lack of evidence of a direct role for cathepsin D in breast cancer cell invasion is consistent with a recent report using the Matrigel invasion assay to study a variety of MCF7 cell clones (Johnson et al, 1993).

Cysteine proteinase inhibitors produced a modest suppression of amnion membrane invasion by metastasizing murine mammary adenocarcinoma cells (Yagel et al, 1989), and increased cathepsin L mRNA in cell lines cloned from a peritoneal murine mammary tumour was associated with increased invasiveness using the matrigel assay (Morris et al, 1993). Furthermore, cathepsin B has recently been shown to be secreted in an active form following intracellular translocation to the cell periphery in three different malignant cell lines, including the c-Ha-*ras*-transfected MCF-10 human breast epithelial cell line (Rozhin et al, 1994). Under similar experimental conditions and concentrations of E64 as in the present study, a substantial reduction of matrigel invasion by ER-negative HOC-1 ovarian cancer cells has been reported (Kobayashi et al, 1992). Although in our study E64 appears to enhance the effect of pepstatin and BB94, the absence of any consistent and major effect of E64 on BMD by MDA-MB-231 or $MCF7_{DOX}$ cells suggests that cysteine proteinases probably play only a minor role in the BM degradation by ER-negative human breast cancer cells. Inhibition of motility of human melanoma and rat carcinosarcoma cells by cysteine proteinase inhibitors, including E64, has been observed (Boike et al, 1991), and this might account for some of the observations with amnion and matrigel models.

In contrast to the minor effects of lysosomal proteinases, inhibitors of uPA or plasminogen activation under SC conditions caused significant reductions in BMD. We have shown directly that the essential serum factor is plasminogen and that inhibition of its conversion to plasmin by uPA with an anticatalytic uPA antibody totally abolishes its effect. As uPA is preferentially activated when bound to its receptor at the cell surface (Ellis and Dano, 1992), the serine proteinase activity on BM might be expected to be largely cell associated, and indeed Yee and Shui (1986) suggested that cell contact was essential for BMD as they observed no activity with SC-CM generated by cell culture on plastic. In our study, SC-CM exhibited appreciable BM degradation that was completely and exclusively inhibited by aprotinin, suggesting that non-cell-associated BM degradation is mediated by the uPA/plasmin system. Interestingly, we observed that, while SC-CM from MDA-MB-231 cells grown on BM had approximately 20% of the BMD activity obtained with the cells, SC-CM from cultures of $MCF7_{DOX}$ cells accounted for almost 80% of the cell-associated activity. This cell line also had a somewhat different inhibitory profile, which might reflect its drug resistance phenotype.

The importance of uPA/plasmin in the in vitro invasion of various non-breast cancer cell lines has been well described (Mignatti et al, 1986; Kobayashi et al, 1992; Reiter et al, 1993). Aprotinin, tranexamic acid and amiloride have all been reported to have antimetastatic effect in vivo, including use in the treatment of cancer patients (Kikuchi et al, 1986, 1987; Kellen et al, 1988; Uetsuji et al, 1992). Although it has been known for some years that MDA-MB-231 cells express much higher levels of uPA than the non-invasive $MCF7_{WT}$ cells (Mangel et al, 1988), we believe the present study is the first to provide direct experimental evidence of a role for uPA in the degradation of BM by breast cancer cells. Our findings are consistent with the recent report of Holst-Hansen et al (1996) in which matrigel invasion by MDA-MB-231 BAG cells was inhibited by antibodies to uPA or uPA receptor (uPAR). These workers also demonstrated that, in contrast to MCF-7 BAG and MDA-MB-435 BAG cells, the MDA-MB-231 BAG cell line expressed high protein levels of uPA and uPAR and was consequently highly active in generating plasmin; the latter being abolished by the uPA and uPAR antibodies. Further study of inhibitors of uPA and plasminogen activation as antimetastatic drugs in the treatment of breast cancer would seem to be warranted.

There is good circumstantial evidence for a potential role for MMPs in breast cancer invasion from both tumour homogenate and immunohistochemical studies (Monteagudo et al, 1990; Davies et al, 1993*a*). Treatment of mice bearing human ovarian cancer xenografts with the broad spectrum MMP inhibitor BB94 (Batimastat) reduced tumour burden and increased survival (Davies et al, 1993*b*). In a more recent study, BB94 reduced the incidence of local tumour recurrence and formation of lung metastases when administered to nude mice after resection of MDA-MB-435 primary tumours (Sledge et al, 1995). This effect was not associated with changes in the tumour expression of MMPs or TIMP-2. We have shown that BB94 significantly reduces MDA-MB-231 cell-associated BMD under both SC and SF conditions. Furthermore, with these cells, the MMP activity appears to be

© Cancer Research Campaign 1997

solely cell-associated, in that we observed no effect of BB94 on BM degradation by SC-CM.

Full details of the different MMPs expressed by these cell lines have yet to be described, and it is possible that one or more species are involved. MDA-MB-231 and $MCF7_{DOX}$ cells have been shown to lack endogenous production of MMP-2 (pro-gelatinase A, 72-kDa type IV collagenase), although they can activate pro-MMP-2 of serum origin trapped in culture matrix containing collagen I (Azzam et al, 1993). However, as BB94 reduced BMD under SF conditions, our data suggests that other MMPs, such as interstitial collagenases or stromelysins, may be involved. However, in tumours, MMPs of stromal origin may play an important role. While TIMP-2 has a preference for complexing with pro-MMP-2, it can inhibit most active MMPs (DeClerck and Imren, 1994). Nevertheless, in this study, TIMP-2 had only a modest inhibitory effect on BMD by MDA-MB-231 cells, particularly in comparison with BB94.

It has been proposed that activation of MMPs is brought about by plasmin, being the final step in a proteolytic cascade (Mignatti et al, 1986; Reich et al, 1988; Schmitt et al, 1992). However, for the breast cancer cells studied, we found little to suggest that the MMP activity was dependent upon uPA/plasmin activation; BB94 reduced 'absolute' BMD values by very similar amounts under SC and SF conditions, and combination with inhibitors of uPA/plasminogen activation, in the presence of serum, had an additive effect. Indeed, the latter observation suggests that MMPs and uPA/plasminogen activation act independently in breaking down BM as others have proposed (Mignatti et al, 1986; Lim et al, 1996).

The relatively small reductions in cell proliferation seen with BB94 and amiloride are probably related to some non-specific cytotoxicity. TIMP-2 produced a similar decrease in cell numbers which might represent a more specific inhibition of cell growth (DeClerck and Imren, 1994), although growth stimulation by TIMP-2 has also been observed (Hayakawa et al, 1994; Nemeth et al, 1996). The profound reduction of cell proliferation with the combination of BB94 and the cysteine proteinase inhibitors E64 or E64d is surprising in view of the minimal effect of the two inhibitors separately. E64d is a membrane-permeant derivative of E64 and is an ethyl ester that is converted under cell culture conditions to the more potent inhibitor Ep475 (Shoji-Kasai et al, 1988). Thus, cell uptake and greater potency of inhibition do not appear to be important factors in this phenomenon. The effects of the combination of BB94 and E64 or E64d may represent stablization of one inhibitor by the other, although BB94 is very stable under the cell culture conditions used (Dr P D Brown, personal communication). E64d has been shown to arrest human epidermoid carcinoma A431 cells at mitotic metaphase (Shoji-Kasai et al, 1988), and it is possible that inhibition of a key cysteine proteinase-dependent process in cell division is in some way enhanced by MMP inhibition. Clearly, further study into the mechanisms involved is required.

In conclusion, inhibitors of uPA/plasminogen activation and MMPs may be of potential therapeutic value in human breast cancer. BB94 and related MMP inhibitors are currently undergoing clinical trials in patients with advanced disease. There is also interest in developing highly specific and sensitive inhibitors of uPA as antimetastatic agents (Towle et al, 1993). Combined inhibition of uPA/plasminogen activation and MMP as antimetastatic therapy for patients with breast cancer deserves clinical evaluation.

### ACKNOWLEDGEMENTS

This work was supported by grants from the Gunnar Nilsson Cancer Research Trust, City Hospital, Dudley Road, Birmingham, and the School of Medicine, University of Birmingham, UK. The authors are grateful to Dr Peter D Brown and Mr Alan Galloway of British Biotech, Oxford, UK, for gifts of BB94 and recombinant TIMP-2. We thank Dr Peter Andreason, Department of Molecular Biology, University of Aarhus, Denmark, for the gift of the anti-uPA antibody, Dr Micheal D Taylor, Department of Chemistry, Parke-Davis Pharmaceutical Research, Ann Arbor, USA, for the gift of PD 134678-0073, and Dr Masaham Tamai, Taisho Pharmaceutical, Research Centre, Saitama, Japan, for the gift of E64d. We thank staff at the Maternity Hospital, Birmingham for the supply of umbilical cords and Mrs Lesley Tomkins, School of Biochemistry, University of Birmingham, for the SEM. We gratefully acknowledge the technical support of Mrs Christine Hail and Mrs Denise Youngs, University Department of Surgery.

### REFERENCES

Alkjaersig N, Fletcher AP and Sol S (1959) ε-Aminocaproic acid: an inhibitor of plasminogen activation. *J Biol Chem* **234**: 832–837

Azzam HS, Arand G, Lippman ME and Thompson EW (1993) Association of MMP-2 activation potential with metastatic progression in human breast cancer cell lines independent of MMP-2 production. *J Natl Cancer Inst* **85**: 1758–1764

Barrett AJ (1977) Cathepsin D and other carboxyl proteinases. In *Proteinases in Mammalian Cells and Tissues*, Barrett AJ (ed.), pp. 209–248. Elsevier/North Holland Biomedical: Amsterdam

Barrett AJ, Kembhavi AA, Brown MA, Kirschke H, Knight CG, Tamai M and Hanada K (1982) L-trans-Epoxysuccinyl-leucylamido(4-guanidino)butane (E-64) and its analogues as inhibitors of cysteine proteinases including cathepsins B, H and L. *Biochem J* **201**: 189–198

Boike G, Lah T, Sloane BF, Rozhin J, Honn K, Guirguis R, Strache ML, Liotta LA and Schiffmann E (1991) A possible role for cysteine proteinases and its inhibitors in motility of malignant melanoma and other tumour cells. *Melanoma Res* **1**: 333–340

Briozzo P, Morrisset F, Capony F, Rougeot C and Rochefort H (1988) In vitro degradation of extracellular matrix with Mr52000 cathepsin D secreted by breast cancer cells. *Cancer Res* **48**: 3688–3692

Brünner N, Boysen B, Romer J and Spang-Thomsen M (1993) The nude mouse as an in vivo model for human breast cancer invasion and metastasis. *Breast Cancer Res Treat* **24**: 257–264

Buck MR, Karustis DG, Day NA, Honn KV and Sloane BF (1992) Degradation of extracellular-matrix proteins by human cathepsin B from normal and tumour tissues. *Biochem J* **282**: 273–278

Davies B, Miles DW, Happerfield LC, Naylor MS, Bobrow LG, Rubens RD and Balkwill FR (1993*a*) Activity of type IV collagenases in benign and malignant breast disease. *Br J Cancer* **67**: 1126–1131

Davies B, Brown PD, East N, Crimmin MJ and Balkwill FR (1993*b*) A synthetic matrix metalloproteinase inhibitor decreases tumor burden and prolongs survival of mice bearing human ovarian carcinoma xenografts. *Cancer Res* **53**: 2087–2091

DeClerck YA and Imren S (1994) Protease inhibitors: role and potential therapeutic use in human cancer. *Eur J Cancer* **30A**: 2170–2180

Doherty AM, Sircar I, Kornberg BE, Quin J, Winters RT, Kaltenbronn JS, Taylor MD, Batley BL, Rapundalo SR, Ryan MJ and Painchaud CA (1992) Design and synthesis of potent, selective, and orally active fluorine-containing renin inhibitors. *J Med Chem* **35**: 2–14

Ellis V and Dano K (1992) The urokinase receptor and the regulation of cell surface plasminogen activation. *Fibrinolysis* **6**: (suppl.4): 27–34

Fidler IJ (1990) Critical factors in the biology of human cancer metastasis: twenty-eighth GHA Clowes Memorial Award Lecture. *Cancer Res* **50**: 6130–6138

Fritz H and Wunderer G (1983) Biochemistry and applications of aprotinin, the kallikrein inhibitor from bovine organs. *Arzneim Forsch Drug Res* **33**: 479–494

Gabrijelcic D, Svetic B, Spaic D, Skrk J, Budihna M, Dolenc I, Popovic T, Cotic V and Turk V (1992) Cathepsins B, H and L in human breast cancer. *Eur J Clin Chem Clin Biochem* **30**: 69–74

© Cancer Research Campaign 1997

Garcia M, Derocq D, Pujol P and Rochefort H (1990) Overexpression of transfected cathepsin D in transformed cells increases their malignant phenotype and metastatic potency. *Oncogene* **5**: 1809–1814

Hayakawa T, Yamashita K, Ohuchi E and Shinagawa (1994) Cell growth-promoting activity of tissue of metalloproteinases-2 (TIMP-2). *J Cell Sci* **107**: 2373–2379

Holst-Hansen C, Johannessen B, Høyer-Hansen G, Rømer J, Ellis V and Brünner N (1996) Urokinase-type plasminogen activation in three human breast cancer cell lines correlates with their in vitro invasiveness. *Clin Exp Metastasis* **14**: 297–307

Isgar B, Jones CE, Stonelake PS, Neoptolemos JP and Baker PR (1991) The effect of proteinase inhibitors on growth and cathepsin D levels of human breast cancer cells. *Br J Cancer* **63**: (suppl. XIII): 21

Johnson MD, Jeffrey AT, Lippman ME and Dickson RB (1993) The role of cathepsin D in the invasiveness of human breast cancer cells. *Cancer Res* **53**: 873–877

Kellen JA, Mirakian A and Kolin A (1988) Antimetastatic effect of amiloride in an animal tumour model. *Anticancer Res* **8**: 1373–1376

Kikuchi Y, Kizawa I, Oomori K, Matsuda M and Kato K (1986) Adjuvant effects of tranexamic acid to chemotherapy in ovarian cancer patients with large amount of ascites. *Acta Obstet Gynecol Scand* **65**: 453–456

Kikuchi Y, Kizawa I, Oomori K, Miyauchi M, Kita T, Sugita M, Tenjin Y and Kato K (1987) Establishment of a human ovarian cancer cell line capable of forming ascites in nude mice and effects of tranexamic acid on cell proliferation and ascites formation. *Cancer Res* **47**: 592–596

Kobayashi H, Ohi H, Sugimura M, Shinohara H, Fujii T and Terao T (1992) Inhibition of in vitro ovarian cancer cell invasion by modulation of urokinase-type plasminogen activator and cathepsin B. *Cancer Res* **52**: 3610–3614

Lim Y-T, Sugiura Y, Laug WE, Sun B, Garcia A and DeClerk YA (1996) Independent regulation of matrix metalloproteinases and plasminogen activators in human fibrosarcoma cells. *J Cell Physiol* **167**: 333–340

Mangel WF, Toledo DL, Nardulli AM, Reiner GCA, Norman MJ and Katzenellenbogen BS (1988) Plasminogen activators in human breast cancer cell lines: hormonal regulation and properties. *J Steroid Biochem* **30**: 79–88

Mignatti P, Robbins E and Rifkin DB (1986) Tumor invasion through the human amniotic membrane: requirement for a proteinase cascade. *Cell* **47**: 487–498

Miles LA and Plow EF (1985) Binding and activation of plasminogen on the platelet surface. *J Biol Chem* **260**: 4303–4311

Minafra S, Morello V, Glorioso F, La Fiura AM, Tomasino RM, Feo S, McIntosh D and Woolley DE (1989) A new cell line (8701-BC) from primary ductal infiltrating carcinoma of human breast. *Br J Cancer* **60**: 185–192

Montcourrier P, Mangeat PH, Salazar G, Morisset, Sahuquet A and Rochefort H (1990) Cathepsin D in breast cancer cells can digest extracellular matrix in large acidic vesicles. *Cancer Res* **50**: 6045–6054

Monteagudo C, Merino MJ, Josefina S-J, Liotta LA and Stetler-Stevenson WG (1990) Immunohistochemical distribution of type IV collagenase in normal, benign and malignant breast tissue. *Am J Pathol* **136**: 585–592

Morris VL, Tuck AB, Wilson SM, Percy D and Chambers AF (1993) Tumor progression and metastasis in murine D2 hyperplastic alveolar nodule mammary tumor cell lines. *Clin Exp Metastasis* **11**: 103–112

Mosquera D, Jones CEB and Goldman MD (1991) The effect in-vitro of harvesting enzymes on endothelial cell adhesion to PTFE. *Surg Res Comm* **11**: 193–199

Nemeth JA, Rafe A, Steiner M and Goolsby CL (1996) TIMP-2 growth-stimulatory activity: a concentration- and cell type-specific response in the presence of insulin. *Exp Cell Res* **224**: 110–115

Reich R, Thompson EW, Iwamoto Y, Martin GR, Deason JR, Fuller GC and Miskin R (1988) Effects of inhibitors of plasminogen activator, serine proteinases and collagenase IV on the invasion of basement membranes by metastatic cells. *Cancer Res* **48**: 3307–3312

Reiter LS, Kruithof EKO, Cajot J-F and Sordat B (1993) The role of the urokinase receptor in extracellular matrix degradation by HT29 human colon cells. *Int J Cancer* **53**: 444–450

Rozhin J, Gomez AP, Ziegler GH, Nelson KK, Chang YS, Fong D, Onoda JM, Honn KV and Sloane BF (1990) Cathepsin B to cysteine proteinase inhibitor balance in metastatic cell subpopulations isolated from murine tumors. *Cancer Res* **50**: 6278–6284

Rozhin J, Sameni M, Ziegler G and Sloane BF (1994) Pericellular pH affects distribution and secretion of cathepsin B in malignant cells. *Cancer Res* **54**: 6517–6525

Schmitt M, Janicke F and Graeff H (1992) Tumor-associated proteases. *Fibrinolysis* **6**: (suppl.4): 3–26

Scragg MA and Ferreira LR (1991) Evaluation of different staining procedures for the quantification of fibroblasts cultured in 96-well plates. *Anal Biochem* **198**: 80–85

Shoji-Kasai Y, Sensho M, Iwashita S and Imahori K (1988) Thiol protease-specific inhibitor E-64 arrests human epidermoid carcinoma A431 cells at mitotic metaphase. *Proc Natl Acad Sci* **85**: 146–150

Sledge GW, Qulali M, Goulet R, Bone EA and Fife R (1995) Effect of matrix metalloproteinase inhibitor Batimastat on breast cancer regrowth and metastasis in athymic mice. *J Natl Cancer Inst* **87**: 1546–1550

Stephens RW, Pollanen J, Tapiovaara H, Leung K-C, Sim P-S, Salonen E-M, Ronne E, Behrendt N, Dano K and Vaheri A (1989) Activation of pro-urokinase and plasminogen on human sarcoma cells: a proteolytic system with surface-bound reactants. *J Cell Biol* **108**: 1987–1995

Thompson EW, Paik S, Brünner N, Sommers CL, Zugmaier G, Clarke R, Shima TB, Torri J, Donahue S, Lippman ME, Martin GR and Dickson RB (1992) Association of increased basement membrane invasiveness with absence of estrogen receptor and expression of vimentin in human breast cancer cell lines. *J Cell Physiol* **150**: 534–544

Towle MJ, Lee A, Maduakor C, Schwartz CE, Bridges AJ and Littlefield BA (1993) Inhibition of urokinase by 4-substituted Benzo[b]thiophene-2-carboxamidines: an important new class of selective urokinase inhibitor. *Cancer Res* **53**: 2553–2559

Uetsuji S, Yamamura M, Takai S, Hioki K and Yamamoto M (1992) Effect of aprotinin on metastasis of Lewis lung tumor in mice. *Jpn J Surg* **22**: 439–442

Vasishta A, Baker PR, Preece PE, Wood RAB and Cushieri A (1988) Inhibition of proteinase-like peptidase activities in serum and tissue from breast cancer patients. *Anticancer Res* **8**: 785–790

Vassalli J-D and Belin D (1987) Amiloride selectively inhibits the urokinase-type plasminogen activator. *FEBS Lett* **214**: 187–191

Yagel S, Warner AH, Nellans HN, Lala PK, Waghorne C and Denhardt DT (1989) Suppression by cathepsin L inhibitors of the invasion of amnion membranes by murine cancer cells. *Cancer Res* **49**: 3553–3557

Yee C and Shui RPC (1986) Degradation of endothelial basement membrane by human breast cancer cell lines. *Cancer Res* **46**: 1835–1839

© Cancer Research Campaign 1997